# Transcriptome Profiling Revealed Light-Mediated Gene Expression Patterns of Plants in Forest Vertical Structures

**DOI:** 10.3390/biology14040434

**Published:** 2025-04-17

**Authors:** Qiming Mei, Yi Zheng, Jiayi Feng, Zhengfeng Wang, Honglin Cao, Juyu Lian

**Affiliations:** 1 Key Laboratory of Vegetation Restoration and Management of Degraded Ecosystems, South China Botanical Garden, Chinese Academy of Sciences, Guangzhou 510650, China; qmei4597@outlook.com (Q.M.); zhengyi17@scbg.ac.cn (Y.Z.); jy-feng@hotmail.com (J.F.); wzf@scib.ac.cn (Z.W.); caohl@scib.ac.cn (H.C.); 2 Center of Plant Ecology, Core Botanical Gardens, Chinese Academy of Sciences, Guangzhou 510650, China; 3 Guangdong Provincial Key Laboratory of Applied Botany, South China Botanical Garden, Chinese Academy of Sciences, Guangzhou 510650, China; 4 Guangzhou Urban Planning & Designing Research Institute Co., Ltd., Guangzhou 510060, China

**Keywords:** expression pattern, photosynthesis, photorespiration, photoreception, photoprotection, forest vertical structure

## Abstract

Light is a critical environmental factor that shapes forest communities. The canopy trees intercept the light, thus understory plants become shaded. Shade leads to the attenuation of light intensity and a shift in the spectrum through the forest vertical structure. The capacity of forest trees to survive and grow under conditions of light heterogeneity is closely related to the intrinsic property of these species. Therefore, identifying how plants interact with light-regime variability is an important research objective of community ecology. In this study, we investigated the light-mediated gene expression patterns in forest vertical structures utilizing transcriptome profiling. The expression levels of 20 annotated genes closely related to photosynthesis, light receptors, and photoprotection were used as traits to estimate how variable light environments influence the plants in forest vertical structures. In summary, the shade-tolerant species were characterized by higher levels of photoreceptor (*phot1/2* and *phyA/B*), photorespiration (*pglp1/2*), and photoprotection genes (*Lhca5*, *Lhca7*, and *PsbS* and photolyases), but with a lower abundance of photosynthetic light-harvesting genes (*Lhca1/2* and *Lhcb1/2*). Also, the expression of light-harvesting and photoprotection genes were generally up-regulated by intense light, while the expression of photoreceptor genes was up-regulated by shade. This research highlights how differential plant responses to light shape the vertical structure of plant communities in a subtropical forest.

## Simple Summary

Light-regime variability is a crucial environmental factor shaping a forest community. Adaptation to light is synergistically regulated by the expression pattern of multiple genes. In this study, we provide several parameters to interpret the light acclimation of plants in a natural subtropical forest, including the expression abundance of genes related to photosynthesis, photosensing, and photoprotection. In summary, the shade-tolerant species are characterized by higher levels of photoreceptor (*phot1/2* and *phyA/B*) and photoprotection genes (*Lhca5*, *Lhca7*, *PsbS*, and photolyases), but with a lower abundance of photosynthetic light-harvesting genes (*Lhca1/2* and *Lhcb1/2*). Also, the expressions of light-harvesting and photoprotection genes were generally up-regulated by intense light, while the expression of photoreceptor genes was up-regulated by shade. This research highlights how differential plant responses to a heterogeneous light environment shape the vertical structure of plant communities in a subtropical forest, offering theoretical foundations for the rehabilitation of regional forest ecosystems.

## 1. Introduction

The coexistence of plant species represents competition for limited environmental resources. For light, the competition is one-sided: taller trees arrogate most of the light energy and shorter individuals are likely to be excluded [1,2]. Here lies the paradox that trees with various statures are usually observed in forests, and understory plants can grow and reproduce in the shade of tall neighbors. The coexistence is a trade-off between growth in full sun and survival in the shade [1]. However, the mechanism underlying the coexistence is yet to be well studied.

Light-regime variability is a crucial environmental factor shaping a forest community [3,4]. In forests, the dense canopy intercepts the radiation, and understory plants become shaded. Shade leads to the attenuation of light intensity and a shift in the spectrum through the forest vertical structure. Plant leaves absorb the light of photosynthetically active radiation (PAR) (400–700 nm), namely ultraviolet-A (UV-A) (320–400 nm) and ultraviolet-B (UV-B) (280–320 nm), and far-red light (700–800 nm) penetrates the canopy filter and is enriched in the canopy shade [5,6]. The capacity of forest trees to survive and grow with different degrees of canopy coverage is closely related to the intrinsic property of the species (i.e., shade-tolerant or light-demanding species) [7].

Light is the main source of energy for photosynthesis. In higher plants, light is captured by two photosystems (PS I and II), which bind photosynthetic pigments such as chlorophyll a, chlorophyll b, and carotenoids [8]. PS I and PS II are large multi-subunit protein complexes assembled by a set of light-harvesting chlorophyll-a/b (LHC) proteins (LHCA1-5 for PS I and LHCB1-7 for PS II) [9,10,11]. Also, RuBisCO (ribulose 1,5-bisphosphate carboxylase/oxygenase, RBC) is a key enzyme in photosynthesis that catalyzes the fixation of CO_2_ [12,13]. The RuBisCO protein is integrated by a large subunit encoded by the *rbcL* gene and a small subunit encoded by the *rbcS* gene [14]. In turn, RuBisCO mediates the oxygenation reaction in the photorespiration pathway that re-releases part of the fixed carbon in photosynthesis [15]. Photorespiration occurs when plants are exposed to stress (e.g., high levels of light), and they dissipate excess energy, thus protecting them against photodamage [16]. Moreover, 2-phosphoglycolate phosphatase (PGLP1 and PGLP2) is an important enzyme that rapidly removes toxic photorespiration metabolites and maintains photosynthesis activity at high light stress [17].

Also, light is an important environmental signal that regulates the growth of plants [18]. In plants, the light-sensing apparatus is facilitated by light receptors including cryptochrome (CRY), phototropin (PHOT), and phytochrome (PHY) [19]. The blue light/UV-A receptor cryptochrome performs essential physiological functions in plants, such as photomorphogenesis, the circadian rhythm, and phototropism [20]. Phototropin is a blue-light receptor regulating phototropism, light-induced stomatal opening, and chloroplast movements of plants [21]. The phytochromes perceive red/far-red light and perform functions in seed germination, the timing of flowering, and the circadian rhythm [22]. The photoreceptors orchestrate the expression pattern of a large number of genes in plants during photomorphogenesis [23]. Moreover, photoreceptors control the responses of plants to avoid exposure to limiting or excessive light conditions, such as shade avoidance syndrome (SAS), thus enhancing plants’ adaptation to unavoidable and unfavorable light environments [5,24,25].

On the other hand, exposure to intense sunlight far above the light saturation point of photosynthesis (high light stress) induces the photodamage of plants through the generation of reactive oxygen species [26,27]. The PSBS (Photosystem II 22 kDa) proteins protect plant photosystems against high light stress by dissipating excess light energy via the regulation of non-photochemical quenching (NPQ) [28,29]. In addition, plants are unavoidably exposed to ultraviolet-B (UV-B) radiation (280–320 nm) which causes DNA damage [30]. Over their long evolutionary history, plants evolved an effective DNA repair system that consists of several subfamilies of photolyase (PHR), including cyclobutane pyrimidine dimer (CPD) photolyase, pyrimidine-pyrimidone (6-4) photolyase, and cryptochrome-DASH (repairing single-strand DNA) [31]. Photolyases are a group of flavoproteins that share sequence similarity with the photoreceptor cryptochrome but perform distinct functions [32].

Functional traits are the morphological, biochemical, physiological, structural, phenological, or behavioral characteristics of organisms that influence performance or fitness [33]. Functional traits of leaves and photosynthesis patterns were shown to closely relate to the forest canopy and vertical structure [34,35]. In the last decade, the rapid development of high-throughput RNA-Seq technology has enabled researchers to address the ecological question regarding how gene expression responds to environmental change [36,37]. For example, Han et al. [38] studied the relationship between seedling mortality and variable light environments by transcriptomes in a subtropical tree community; Swenson et al. [39] reported that species exhibiting similar transcriptomic profiles in their response to drought tend to non-randomly co-occur. In this study, we investigated how canopy and understory trees respond to heterogeneous light environments by transcriptome profiling analysis and elucidated the mechanism underlying the species coexistence along with the vertical structure in a mature subtropical evergreen forest. Specifically, we address two questions: (1) How do light-demanding and shade-tolerant species differ in their transcriptome profiles? (2) How do species respond to different light environments (the top vs. bottom of canopies)?

## 2. Materials and Methods

### 2.1. Study Site and Plant Material

**Figure 1:**
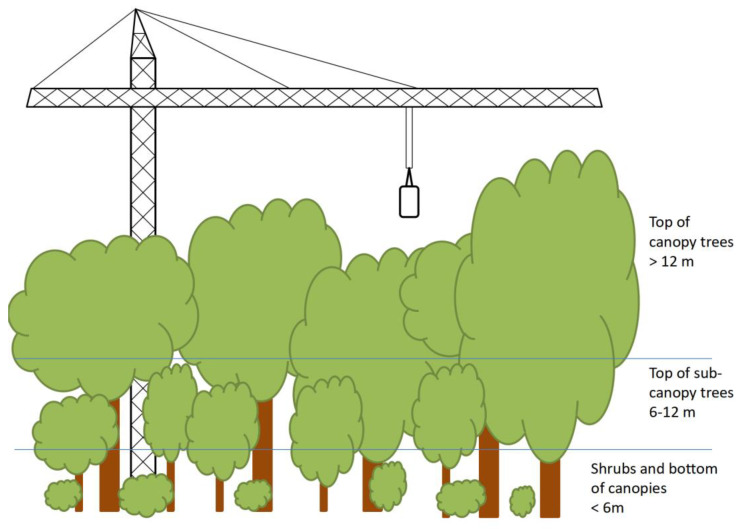
Forest canopy layers and sampling positions.

The study site was located in the Dinghushan National Nature Reserve (23°09′21″–23°11′30″ N, 112°30′30″–112°33′41″ E), Zhaoqing City, Guangdong Province, China. The most abundant woody species within the studied forest were involved in this analysis. Fresh leaves were collected from four canopy trees (*Castanopsis chinensis*, *Cryptocarya chinensis*, *Machilus chinensis*, and *Schima superba*), four sub-canopy trees (*Aporosa dioica*, *Cryptocarya concinna*, *Schefflera octophylla*, and *Sterculia lanceolata*), an understory shrub (*Psychotria rubra*), and a sapling of a pioneer tree species, which only occurred in the forest gap (*Castanopsis fissa*), in August 2019. We used a tower crane to reach the top of the canopy over 12-meter (Figure 1). For dominant and understory trees, the leaves of three individuals were collected from the top and bottom of the canopy; for *P. rubra* and *C. fissa*, one leaf sample from each individual (3 individuals were used in this study as biological replicates) was collected. Then, leaf tissues were frozen immediately in liquid nitrogen and stored at −80 °C in a freezer (Thermo Fisher Scientific, Waltham, MA, USA).

### 2.2. RNA Extraction and RNA-Seq

About 80 mg of tissue from each sample was used for RNA extraction with the CTAB-pBIOZOL reagent (Bioer, Hangzhou, China). Then, total RNA was qualified by a NanoDrop (Thermo Fisher Scientific, Waltham, MA, USA) and quantified using an Agilent 2100 bioanalyzer (Agilent, Santa Clara, CA, USA). The mRNA was purified using oligo (dT)-attached magnetic beads (NEB, Ipswich, MA, USA). Next, the mRNA was cleaved into small fragments using divalent cations under elevated temperatures. Then, the first and second strands of cDNA were synthesized using reverse transcriptase (Thermo Fisher Scientific, Waltham, MA, USA) and random primers (Thermo Fisher Scientific, Waltham, MA, USA). A-Tailing Mix and RNA Index Adapters (Takara, Dalian, China) were added, and the cDNA products were purified and enriched with PCR amplification (Thermo Fisher Scientific, Waltham, MA, USA) and AMPure XP Beads (Beckman Coulter, Brea, CA, USA). The PCR products were validated on an Agilent 2100 bioanalyzer (Agilent, Santa Clara, CA, USA) for quality control. The PCR products from the previous step were heated, denatured, and circularized by the splint oligo sequence to generate libraries (formed by single-strand circle (ssCir) DNA). The libraries were amplified with phi29 DNA Polymerase (NEB, Ipswich, MA, USA) to produce the DNA nanoball (DNB), and DNB-based nanoarrays were sequenced on the DNBSEQ platform (BGI, Wuhan, China). Around 2.5 Gb of sequencing data was generated for each sample.

### 2.3. Transcriptome Assembly, Gene Annotation, and Differential Expression Analysis

Clean data were generated by removing reads containing an adapter and those that were of low quality (with a low-quality base ratio ≥ 20% and unknown base ratio ≥ 5%) from the raw data using SOAPnuke v.1.5.2 [40]. Then, Trinity v.2.0.6 was used for assembly of the clean reads [41]. Tgicl v2.0.6 was utilized to eliminate redundant data in the assembled transcripts to obtain unique genes [42].

Clean reads were aligned against the unique gene set utilizing Bowtie2 v.2.2.5 [43]. Genes were annotated by mapping to databases including GenBank databases, including the Nucleotide Sequence Database (NT), Non-Redundant Protein Sequence Database (NR), and KOG (Eukaryotic Orthologous Groups of proteins) using BLAST v.2.2.23 with an e-score ≤ 10^−5^ [44]. Also, GO (Gene Ontology) and KEGG (Kyoto Encyclopedia of Genes and Genomes) annotation was conducted by Blast2GO v.2.5.0 [45]. The expression level of genes was calculated by RSEM v.1.2.8 [46]. Gene expression levels were calculated as FPKM (Fragments Per Kilobase of exon model per Million mapped fragments).

### 2.4. Statistical Analysis

The statistical significance of FPKM values between individuals, species, and canopy layers was estimated by ANOVA using R v.4.1.0 [47]. Firstly, the data were log-transformed to meet normality before the following analysis. Then, we used the pairwise t-test to check the inter-layer (top and bottom) expression differences in each concerned gene for each of the studied species, except *P. rubra* and *C. fissa*, whose leaves were collected from only one canopy layer. In order to check the universality of these inter-layer differences in the gene expression of each forest stratum (canopy or understory), we separately performed linear mixed-effect models where species were treated as the random-effects term [48,49]. We used nested ANOVA for the studied genes one by one to check whether their expression showed a difference between the two strata.

## 3. Results

### 3.1. Expression Pattern of Photosynthetic and Photorespiratory Genes

In plants, light-harvesting complexes I and II (formed by LHCA and LHCB proteins) are responsible for light absorption, and they trigger light reactions of photosynthesis [9]. In this study, we estimated the expression pattern of *Lhc* genes by transcriptome profiling. The *Lhc* genes are classified into two groups based on their expression patterns, including abundantly expressed *Lhca1-4* and *Lhcb1-6*, and rarely expressed *Lhca5* and *Lhcb7* [9]. In this study, abundantly expressed Lhcs were represented by *Lhca1/2* and *Lhcb1/2*.

**Figure 2:**
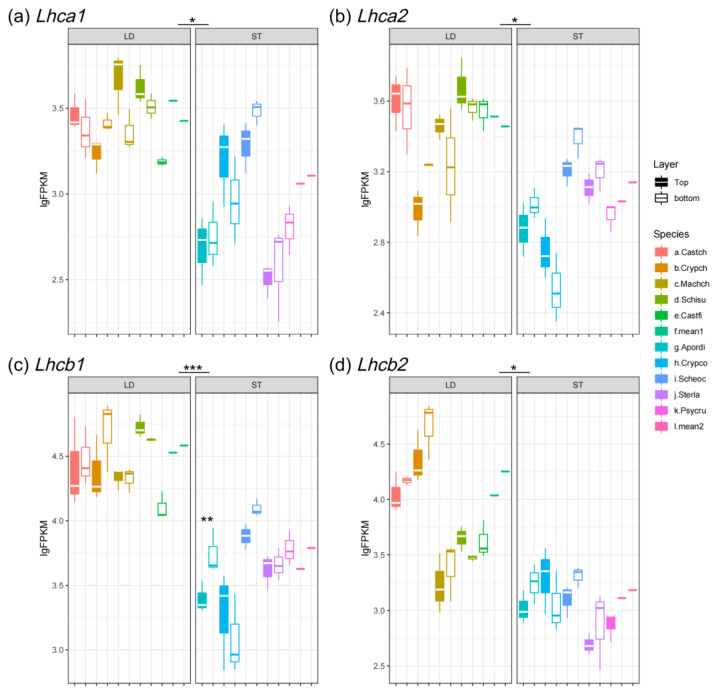
Expression level of abundantly expressed *Lhcs*: (**a**) *Lhca1*; (**b**) *Lhca2*; (**c**) *Lhcb1;* and (**d**) *Lhcb2*. LD represents light-demanding species and ST represents shade-tolerant species. The asterisk symbols indicate the statistical significance: *** *p* < 0.001, ** *p* < 0.01, and * *p* < 0.05.

**Figure 3:**
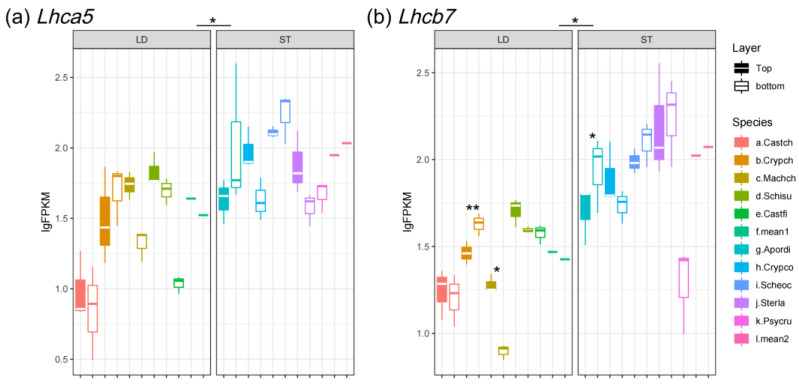
Expression level of rarely expressed *Lhcs*: (**a**) *Lhca5* and (**b**) *Lhcb7*. LD represents light-demanding species and ST represents shade-tolerant species. The asterisk symbols indicate the statistical significance: ** *p* ≤ 0.01, and * *p* ≤ 0.05.

Generally, the expression of light-harvesting genes varied widely between light-demanding and shade-tolerant species. The *Lhca1/2* abundance of light-demanding trees was 3-fold higher as compared to understory trees and the *Lhcb1/2* of light-demanding trees was about 10-fold higher than for understory trees (Figure 2). For example, the mean FPKM value of *Lhcb1* at the bottom of the canopy for light-demanding species was 38,349.87 (lgFPKM = 4.58), while the value for shade-tolerant species was 6138.44 (lgFPKM = 3.79) (Figure 2c). On the other hand, the rarely expressed *Lhca*5 and *Lhcb*7 demonstrated inverse expression patterns to abundantly expressed *Lhcs*: they were significantly more abundant in shade-tolerant species as compared to light-demanding species (Figure 3a,b).

For most of the studied light-demanding species, the FPKM results indicated that the expression of *Lhca1/2* was increased with the higher light intensity (canopy top) (Figure 2a,b). But, for *Lhcb1/2*, the abundance increased in the shade (canopy bottom), e.g., the bottom canopies of *Cryptocarya chinensis* showed the strongest expression of *Lhcb1* and *Lhcb2* (lgFPKM values were 4.75 and 4.71, respectively) (Figure 2c,d). On the other hand, spatial expression patterns of the *Lhca* and *Lhcb* genes of understory species demonstrated similar expression patterns: expression levels were higher at lower canopy heights (Figure 2a–d).

**Figure 4:**
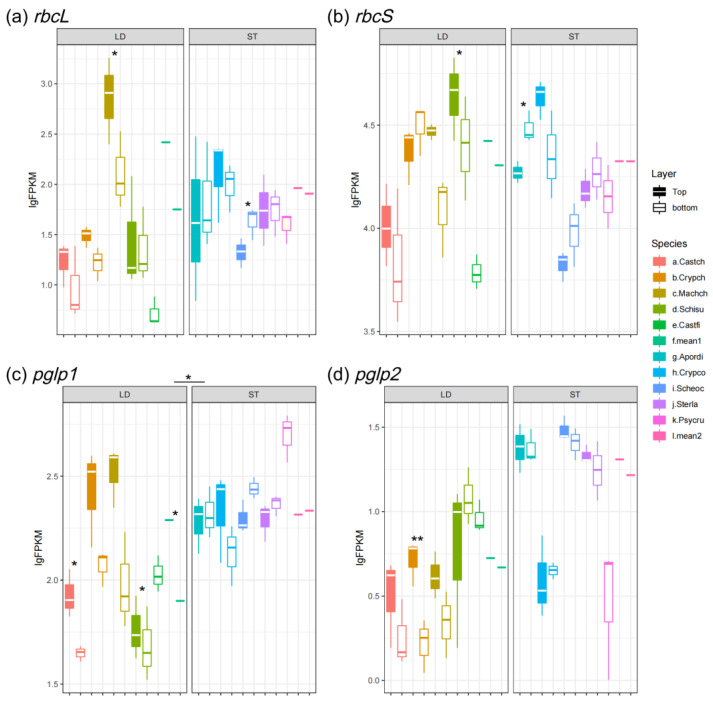
Expression level of carbon fixation and photorespiration genes: (**a**) *rbcL*; (**b**) *rbcS*; (**c**) *pglp1;* and (**d**) *pglp2*. LD represents light-demanding species, and ST represents shade-tolerant species. The asterisk symbols indicate the statistical significance: ** *p* ≤ 0.01, and * *p* ≤ 0.05.

As is shown in Figure 4a,b, the genes coding the subunits of RuBisCO (*rbcL* and *rbcS*) in light-demanding species showed inter-layer differences, e.g., the expression level of *rbcL* in light-demanding trees in the upper canopy layer (FPKM = 261.86, lgFPKM = 2.42) was almost 5-fold higher as compared to the lower canopy layer (FPKM = 56.30, lgFPKM = 1.75). But, for shade-tolerant species, the expression levels of *rbcL* and *rbcS* between the upper and lower canopies were not significantly different (Figure 4a,b). Moreover, the *rbcS* gene of light-demanding and shade-tolerant species exhibited similar levels of expression (Figure 4b).

In addition, we estimated the expression abundance of *pglp1/2* to assess the level of photorespiration. The upper canopy of light-demanding species expressed a much higher level of *pglp1* as compared to the lower canopy (Figure 4c). On the other hand, shade-tolerant species showed a higher level of *pglp1/2*, though they existed in the shade (Figure 4c,d).

### 3.2. Expression Pattern of Photoreceptors

The photoreceptors sensed blue-light-/UV-A- and red/far-red-light-mediated and -controlled phototropism, photomorphogenesis, and circadian rhythms in plants [22,50]. In this study, we estimated how plants respond to various light qualities by analyzing the expression patterns of multiple plant photoreceptors including *cry*, *phot*, and *phy*.

**Figure 5:**
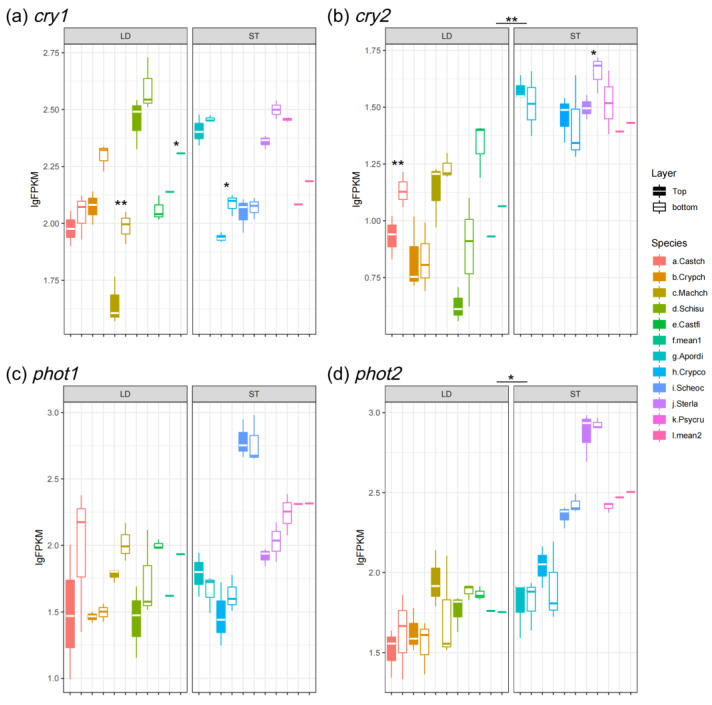
Expression level of blue-light receptors: (**a**) *cry1*; (**b**) *cry2*; (**c**) *phot1*; and (**d**) *phot2*. LD represents light-demanding species and ST represents shade-tolerant species. The asterisk symbols indicate the statistical significance: ** *p* ≤ 0.01, and * *p* ≤ 0.05.

**Figure 6:**
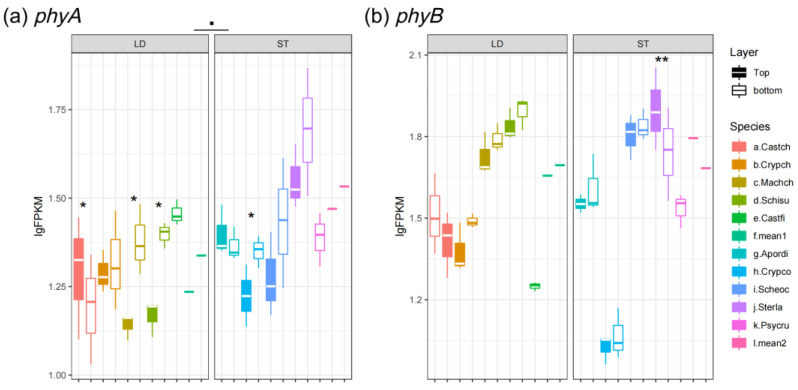
Expression level of red-light receptors: (**a**) *phyA* and (**b**) *phyB*. LD represents light-demanding species and ST represents shade-tolerant species. The asterisk symbols indicate the statistical significance: ** *p* ≤ 0.01, and * *p* ≤ 0.05.

As is shown in Figure 5a,b, the blue light/UV-A receptor *cry1* slightly increased at the bottom of canopies for both light-demanding and shade-tolerant species. But, the expression levels of *cry1* between light-demanding and shade-tolerant species showed no significantly different (Figure 5a). On the other hand, *cry2*, which showed functional redundancy of *cry1*, was more abundant in shade-tolerant species except for *C. concinna* (Figure 5b). For the other family of blue-light photoreceptors, phototropin (including *pot1* and *phot2*) manifested higher expression levels in understory species, and the expression level of *pot1* in light-demanding species was up-regulated at the bottom as compared to the top of canopies (Figure 5c,d). Moreover, the expression of the red-/far-red-light receptor *phyA* was significantly higher in shade-tolerant species, whereas the homolog *phyB* showed no significant difference between layers and species (Figure 6a,b).

### 3.3. Expression Pattern of Photoprotection Gene and Photolyases

**Figure 7:**
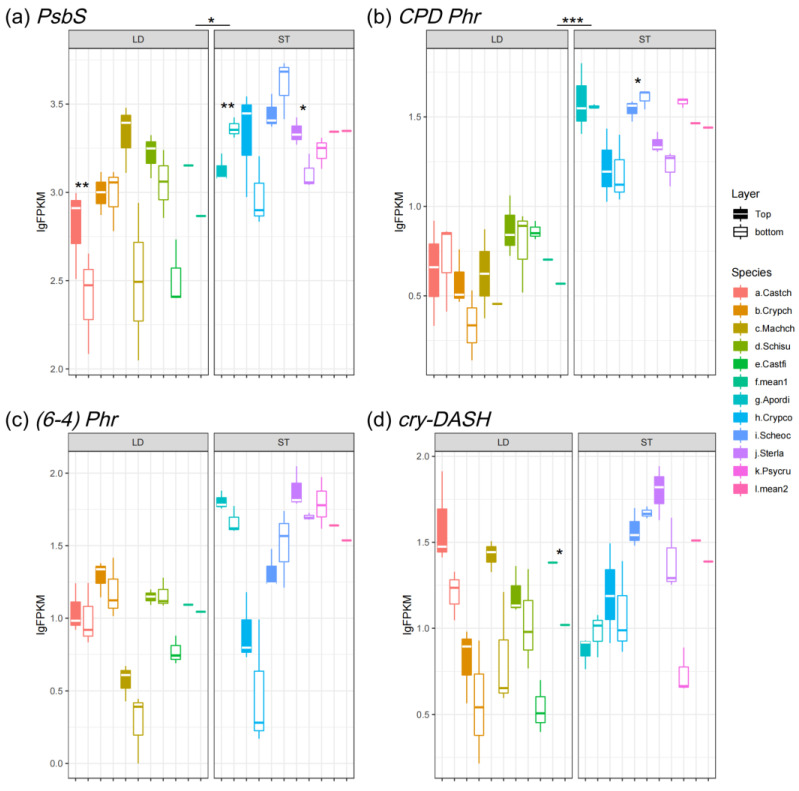
Expression level of photoprotection genes: (**a**) *PsbS*; (**b**) *CPD Phr*; (**c**) *(6-4) Phr*; and (**d**) *cry-DASH*. LD represents light-demanding species and ST represents shade-tolerant species. The asterisk symbols indicate the statistical significance: *** *p* ≤ 0.001, ** *p* ≤ 0.01, and * *p* ≤ 0.05.

Plants protect themselves under high-light-stress conditions by NPQ that dissipates excessive light energy as heat, where progress requires the protein PSBS [51]. *PsbS* was shown to be light-induced and the expression of the gene was up-regulated at the top of canopies and down-regulated in the shade (Figure 7a). Although light-demanding species were exposed to an environment with much stronger irradiation, they showed a lower expression level of *PsbS* than shade-tolerant species (Figure 7a).

Strikingly, shade-tolerant species exhibited higher expression levels of photolyases than light-demanding species, indicating that understory species were more vulnerable to UV radiation (Figure 7b–d). In addition, only *cry-DASH* of light-demanding trees showed an inter-layer difference; expression at the bottom of canopies was restrained (Figure 7d).

## 4. Discussion

Demonstrating how plants in forest structures acclimate to vertical light heterogeneity is an important issue of community ecology, which will help us to understand the coexistence of plant species. Changes in the environmental light intensity and spectrum leads to rapid alterations in gene expression, thus dramatically modifying plants’ morphology and physiology [52]. In this study, we conducted a global transcriptome analysis of 10 woody plants belonging to different canopy layers of a subtropical forest community. Expression levels of 24 annotated genes with functions in photosynthesis, light sensing, and photoprotection, including genes coding six LHCs, two subunits of RuBisCO, two PGLPs, two CRYs, two PHOTs, two PHYs, and three PHRs proteins, were utilized as traits to investigate how variable light environments influence the plants in a forest vertical structure.

Among all of the studied genes, the abundantly expressed *Lhcs* (*Lhca1/2* and *Lhcb1/2*) and *rbcS*, which related to photosynthesis, exhibited the highest levels of expression (Figure 2a–d and Figure 4b). Although the shaded leaves of light-demanding and shade-tolerant species grow in similar light environments, the expression levels of *Lhca1/2* and *Lhcb1/2* are dramatically different (Figure 2a–d). Also, for the light-demanding species, the *rbcS* and *rbcL* genes of the sun-exposed leaves were significantly higher than the shaded leaves (Figure 4a,b). These results are consistent with those previously reported for a light-demanding species, *Eucalyptus globulus*: when compared to middle- and bottom-canopy leaves, the RuBisCO concentration was higher and mRNA levels of RuBisCO-coding genes were up-regulated in the top leaves [53]. Also, *rbcS* and *rbcL* in shade-tolerant species were slightly higher than in light-demanding species at the bottom of the canopy (Figure 4a,b). Previous studies have revealed that shade-tolerant species have lower light compensation points (LCPs) as compared to light-demanding species [54,55]. In addition, the light saturation of photosynthetic CO_2_ fixation decreases as environmental light intensity decreases [56]. The low LCP and light saturation allow shade-tolerant species to use light energy and accumulate carbon more efficiently at a lower light intensity, thus requiring lower levels of *Lhcs* expression but having a higher content of RuBisCO [54]. Generally, canopy trees cannot utilize strong light efficiently because of photosynthetic rate saturation, but the leaves of understory species can use diffuse light efficiently for photosynthesis [2].

On the other hand, photorespiration accompanies photosynthesis under high levels of light. RuBisCO catalyzes two competing reactions in photosynthesis and photorespiration [15]. Therefore, we estimated the expression level of another enzyme in photorespiration—*pglp1/2* (Figure 4c,d). Surprisingly, the expression levels of *pglp1/2* are significantly higher in shade-tolerant species as compared to light-demanding species. These results indicate that although understory species grow in the shade, photosynthesis may also be inhibited by excessive diffuse light and high temperatures. Also, a recent study reported that the high expression level of PGLP enhanced the plants’ ability to accumulating carbon in high levels of light and high temperatures [17].

Although the characteristics of acclimation to light vary greatly among plant species, the spatial expression patterns of *Lhca* and *rbcS* are generally up-regulated by light intensity [57,58]. But, in this case, the expression of *Lhca1/2* by *Cryptocarya chinensis* showed a retrograde pattern under direct sunlight, which is distinct from other light-demanding species (Figure 2a,b). *C. chinensis* may possess a lower light saturation point as compared to other light-demanding species where full sun inhibits photosynthesis. Moreover, the closely related species of *C. chinensis, C. concinna*, exhibits an expression pattern of *Lhca1/2* and *rbcS*, which is in contrast to *C. chinensis* but quite similar to light-demanding species (Figure 2a,b and Figure 4b). *Lhca1-2* and *rbcS* were more abundant in the top leaves of *C. concinna* canopy (Figure 2a,b and Figure 4b). Although the two *Cryptocarya* species were located at separate light niches, we hypothesized that both of them were mid-succession species; *C. concinna* can tolerate shade at the sapling phase and ultimately reaches the upper canopy like *C. chinensis* during the succession of forests.

In general, *Lhcb* genes are most strongly expressed in low light conditions and reduced by high light stress [59,60]. In this study, we found that the *Lhcb1/2* gene of most light-demanding and shade-tolerant species slightly increased in the shade (Figure 2c,d). It has previously been reported that LHCB proteins act as antennas of PS I in the dark [61]. Therefore, LHCB1-2 may take over the light-harvesting function of LHCAs to strengthen the photosynthetic activity under low light conditions when LHCAs are down-regulated.

The rarely expressed *Lhc* genes (*Lhca5* and *Lhcb7*) are more abundant in shade-tolerant species than light-demanding species (Figure 3a,b). The patterns of rarely expressed *Lhcs* are dramatically different from abundantly expressed *Lhcs*, but quite similar to that of *PsbS* (Figure 7a). The rarely and abundantly expressed LHC proteins perform distinct physiological functions [9]. Previous studies have reported that LHCA5 functions in PS I in low light conditions, when other LHCA proteins are less abundant [62], and it is involved in chlororespiration under stress conditions, e.g., high light stress [63]. LHB7 performs NPQ functions to dissipate excess energy when the absorbed light exceeds the electron transfer capacities of the thylakoid complexes [64]. To conclude, there is a functional link between the light harvesting and light protection conducted by the rarely expressed *Lhc* genes.

In plants and other photosynthetic organisms, the LHC proteins fuel photosynthesis by absorbing light energy, whereas the photoreceptors are activated by the different wavelengths of the light spectrum to trigger physiological functions, such as circadian rhythms and SAS [65]. Expression of photoreceptors including *cry*, *phot*, and *phy* are urged under low light conditions to trigger SAS in plants (Figure 5 and Figure 6) [5]. Also, our results demonstrated that the blue-light receptors *cry1* and *pot1* manifested reverse expression patterns as compared to *Lhca1/2* (Figure 2a,b and Figure 5a,c). Plants decrease their photosynthetic activity in a high-light-stress environment, named photoinhibition. The molecular link between photoreception and photoinhibition has been unveiled: the expression level of *Lhc* mRNA is down-regulated and NPQ is activated by photoreceptors [66,67,68]. On the other hand, *cry1*, *pot1*, and *phyA* of both light-demanding and shade-tolerant species exhibit similar levels of abundance (Figure 5a,c and Figure 6a), whereas the homologous genes *cry2*, *phot2*, and *phyB* are significantly different between light-demanding and shade-tolerant species (Figure 5b,d and Figure 6b). The homologous photoreceptors (i.e., *cry1*/*2*, *phot1/2* and *phyA/B*) are functionally redundant [69,70,71]. There is a functional compensation when one of the homologs is at a low level of expression, e.g., *A. dioica* has a relatively low expression of *cry1*, but *cry2* is higher than in other shade-tolerant species (Figure 5a,b).

It seems that the expression level of *PsbS* does not directly reflect the photoprotection ability, because the expression levels are significantly higher in shade-tolerant species, which was observed in low-light-level environments (Figure 7a). Photoinhibition occurs when the extent of photodamage overwhelms the ability of its repair, thus reflecting the balance between photodamage and photoprotection [26]. As a result, the expression of *Lhcbs* should be considered when discussing the capability of photoprotection. For example, the typical light-demanding species *C. fissa* manifests a low level of *PsbS* but a high level of Lhcb1, whereas the understory shrub *P. rubra* exhibits reversal patterns (Figure 3c and Figure 7a).

Also, we found that the three classes of photolyases (CPD PHR, (6-4) PHR, and CRY-DASH) were more abundant in shade-tolerant species than light-demanding species (Figure 7b–d). To maintain photosynthetic activity in low light conditions, shade-tolerant species tend to have larger and thinner leaves [72]. Therefore, the leaves of shade-tolerant species may be more vulnerable to UV-B radiation as compared to light-demanding species. In other words, light-demanding species can survive in high-light-stress conditions with lower expression levels of photolyases.

## 5. Conclusions

Adapting to light is synergistically regulated by the expression pattern of multiple genes. In this study, we provide several parameters to interpret the light acclimation of plants in a natural forest community, including the expression abundance of genes related to photosynthesis, photosensing, and photoprotection. In summary, the shade-tolerant species are characterized by higher levels of photoreceptor (*phot1/2* and *phyA/B*) and photoprotection genes (*Lhca5*, *Lhca7*, *PsbS*, and photolyases), but with a lower abundance of photosynthetic light-harvesting genes (*Lhca1/2* and *Lhcb1/2*) (Table 1). Moreover, light variability influences the plants’ defense system: shade increases the herbivory of insects and infection by pathogens, thus stimulating the defense response of plants [73]. The assembly of a forest’s vertical structure is complex and influenced by more than just light.

So far, we have several technical issues to resolve in this study. RNA-seq provides a broad scope and genes express differently under conditions of environmental heterogeneity. However, the expression levels need to be further validated by other methods such as RT-qPCR and Western blotting.

## Figures and Tables

**Table 1:** Different expression patterns of light-harvesting genes, photoreceptors, and photoprotection genes between light-demanding species and shade-tolerant species.

Genes	Function	Expression Level in Light-Demanding Species	Expression Level in Shade-Tolerant Species
Abundantly expressed LHCs (*Lhca1/2* and *Lhcb1/2*)	Light-harvesting of photosynthesis	Higher	Lower
Rarely expressed LHCs (*Lhca5* and *Lhcb7*)	Light-harvesting of photosynthesis and photoprotection	Lower	Higher
RuBisCO (*rbcL* and *rbcS*)	Carbon fixation and photorespiration	Similar	Similar
2-PG phosphatase (*pglp1/2*)	Photorespiration	Lower	Higher
Cryptochromes (*cry1*/*2*)	Blue- and UV-A-light receptors	Similar	Similar
Phototropins (*pot1*/*2*)	Blue-light receptors	Lower	Higher
Phytochromes (*phyA*/*B*)	Red- and far-red-light receptors	Lower	Higher
NPQ (*PsbS*)	Photoprotection	Lower	Higher
Photolyases (*CPD Phr*, *(6-4) Phr* and *cry-DASH*)	Repairing UV-induced damage	Lower	Higher

## Data Availability

The raw sequencing data were deposited in the NCBI Sequence Read Archive; accession numbers are listed in the Supplementary Materials in Table S1.

## Supplementary Materials

The following supporting information can be downloaded at: https://www.mdpi.com/article/10.3390/biology14040434/s1, Table S1: NCBI accession numbers of transcriptomes used in this study.
