# Supplementary material for: Transcriptome Profiling Revealed Light-Mediated Gene Expression Patterns of Plants in Forest Vertical Structures
Source: Biology (Basel). 2025 Apr 17;14(4):434. doi: 10.3390/biology14040434 (PMC12024868; doi:10.3390/biology14040434)
Supplement: Supplementary file 1 [file biology-14-00434-s001.zip › biology-3517735-supplementary.pdf]

**Table S1.** NCBI accession numbers of transcriptomes used in this study.

| Species | Sample | Height above ground (m) | NCBI accession number |
|---|---|---|---|
| *Aporosa dioica* | Apordi_08_034_1 | 10 | SAMN20718188 |
| | Apordi_08_034_2 | 4.3 | SAMN20718189 |
| | Apordi_10_002_1 | 9 | SAMN20718190 |
| | Apordi_10_002_2 | 5 | SAMN20718191 |
| | Apordi_27_064_1 | 11.3 | SAMN20718192 |
| | Apordi_27_064_2 | 1.8 | SAMN20718193 |
| *Castanopsis chinensis* | Castch_07_056_0 | 21.6 | SAMN20717986 |
| | Castch_07_056_2 | 5.5 | SAMN20717987 |
| | Castch_09_005_0 | 15.5 | SAMN20717988 |
| | Castch_09_005_2 | 2.2 | SAMN20717989 |
| | Castch_15_010_0 | 13.8 | SAMN20717990 |
| | Castch_15_010_2 | 4.5 | SAMN20717991 |
| *Castanopsis fissa* | Castfi_19_004_2 | 1.5 | SAMN20717992 |
| | Castfi_19_039_2 | 1.9 | SAMN20717993 |
| | Castfi_19_040_2 | 2 | SAMN20717994 |
| *Cryptocarya chinensis* | Crypch_09_033_0 | 16.5 | SAMN20717995 |
| | Crypch_09_033_2 | 2.5 | SAMN20717996 |
| | Crypch_23_092_0 | 12 | SAMN20717997 |
| | Crypch_23_092_2 | 4.2 | SAMN20717998 |
| | Crypch_28_042_0 | 18.5 | SAMN20717999 |
| | Crypch_28_042_2 | 5 | SAMN20718000 |
| *Cryptocarya concinna* | Crypco_09_012_1 | 6 | SAMN20718194 |
| | Crypco_09_012_2 | 2.6 | SAMN20718195 |
| | Crypco_09_017_1 | 8 | SAMN20718196 |
| | Crypco_09_017_2 | 1.8 | SAMN20718197 |
| | Crypco_09_021_1 | 6 | SAMN20718198 |
| | Crypco_09_021_2 | 2.3 | SAMN20718199 |
| *Machilus chinensis* | Machch_03_088_0 | 20 | SAMN20718001 |
| | Machch_03_088_2 | 6 | SAMN20718002 |
| | Machch_10_013_0 | 14.5 | SAMN20718003 |
| | Machch_10_013_2 | 3 | SAMN20718004 |
| | Machch_22_035_0 | 21.8 | SAMN20718005 |
| | Machch_22_035_2 | 6 | SAMN20718006 |
| *Psychotria rubra* | Psycru_16_011_2 | 1.5 | SAMN20718200 |
| | Psycru_26_127_2 | 2 | SAMN20718201 |
| | Psycru_21_147_2 | 2 | SAMN20718202 |
| *Schefflera octophylla* | Scheoc_04_069_1 | 7.2 | SAMN20718203 |
| | Scheoc_04_069_2 | 3 | SAMN20718204 |
| | Scheoc_10_016_1 | 8.5 | SAMN20718205 |
| | Scheoc_10_016_2 | 4 | SAMN20718206 |

| | | | |
|---|---|---|---|
| | Scheoc_19_003_1 | 6.5 | SAMN20718207 |
| | Scheoc_19_003_2 | 1.5 | SAMN20718208 |
| *Schima superba* | Schisu_30_001_0 | 13.2 | SAMN20718007 |
| | Schisu_30_001_2 | 6 | SAMN20718008 |
| | Schisu_28_022_0 | 22.8 | SAMN20718009 |
| | Schisu_28_022_2 | 1.4 | SAMN20718010 |
| | Schisu_28_026_0 | 23.1 | SAMN20718011 |
| | Schisu_28_026_2 | 5 | SAMN20718012 |
| *Sterculia lanceolata* | Sterla_04_080_1 | 8.5 | SAMN20718209 |
| | Sterla_04_080_2 | 3.5 | SAMN20718210 |
| | Sterla_09_031_1 | 6.5 | SAMN20718211 |
| | Sterla_09_031_2 | 3.2 | SAMN20718212 |
| | Sterla_10_012_1 | 9.5 | SAMN20718213 |
| | Sterla_10_012_2 | 2.6 | SAMN20718214 |
